# Mechanisms linking gut microbiota to depression

**DOI:** 10.1192/j.eurpsy.2021.1962

**Published:** 2021-08-13

**Authors:** A. Fraga, D. Esteves-Sousa, J. Facucho-Oliveira, M. Albuquerque, M. Costa, P. Espada-Santos, N. Moura, A. Moutinho

**Affiliations:** 1 Psychiatry, Hospital de Cascais, Cascais, Portugal; 2 Psychiatry Department, Ocidental Lisbon Hospital Center, Lisboa, Portugal

**Keywords:** Gut-brain axis, Gut Microbiota, Depression

## Abstract

**Introduction:**

The gut microbiota constitute the largest and most diverse community in the body which is primarily responsible for the maintenance of the intestinal wall integrity and the protection against pathogens. Besides having an important role in the regulation of host energy metabolism, the gut microbiota can also influence neurodevelopment, modulate behavioral and might contribute to the development of psychiatry disorders.

**Objectives:**

The authors elaborated a narrative literature review to understand how gut microbiota can influence depression.

**Methods:**

Using PubMed as the database, a research was conducted about how Gut Microbiota relates with Depression.

**Results:**

The microbiota-gut-brain axis encompasses the strong bidirectional communication between the gut microbiota and the CNS. Multiple mechanisms may be involved in this bilateral communication, including immune, endocrine and neural pathways. Permutations in the gut microbiome composition trigger microbial lipopolysaccharides production that activates inflammatory responses. Cytokines send signals to the vagus nerve, which links the process to the hypothalamic-pituitary-adrenal axis that consequently causes behavioral effects. Beyond this, gut microbiota have the capacity to produce many neurotransmitters and neuromodulators such as serotonin and can induce the secretion of the brain-derived neurotrophic factor, an important plasticity-related protein that promotes neuronal growth, development and survival.

**Conclusions:**

Neuroinflammatory processes like those that occur in depression are deeply modulated by peripheral inflammatory stimuli, especially those from the intestinal microbiota. However, the knowledge is currently limited and the information available is not enough to understand the exact mechanisms. Therefore, more studies are required to show how gut microbiota influences the human brain.

**Disclosure:**

No significant relationships.

